# The tumor inflammation signature (TIS) is associated with anti-PD-1 treatment benefit in the CERTIM pan-cancer cohort

**DOI:** 10.1186/s12967-019-2100-3

**Published:** 2019-11-04

**Authors:** Diane Damotte, Sarah Warren, Jennifer Arrondeau, Pascaline Boudou-Rouquette, Audrey Mansuet-Lupo, Jérôme Biton, Hanane Ouakrim, Marco Alifano, Claire Gervais, Audrey Bellesoeur, Nora Kramkimel, Camille Tlemsani, Barbara Burroni, Angéline Duche, Franck Letourneur, Han Si, Rebecca Halpin, Todd Creasy, Ronald Herbst, Xing Ren, Pascale Morel, Alessandra Cesano, François Goldwasser, Karen Leroy

**Affiliations:** 1 Team Cancer, Immune Control and Escape, Cordeliers Research Center, UMRS 1138, Institut National de la Santé et de la Recherche Médicale (INSERM), Paris, France; 2 University Paris Descartes, Paris, France; 3 Department of Pathology, Hôpital Cochin, AP-HP, Paris, France; 4 CERTIM, Hôpital Cochin, APHP, Paris, France; 5 NanoString Technologies, Seattle, WA USA; 6 Department of Medical Oncology, Hôpital Cochin, AP-HP, Paris, France; 7 Team Physiopathologie, cibles et thérapies de la polyarthrite rhumatoide Laboratoire Immunologie et Immunopathologie-Li2P, UMR1125, Université Paris 13, Bobigny, France; 8 Department of Thoracic Surgery, Hôpital Cochin, AP-HP, Paris, France; 9 Department of Cutaneous Diseases, Hôpital Cochin, AP-HP, Paris, France; 10 Genomic platform, INSERM U1016, Institut Cochin, Paris, France; 11 Oncology Research, MedImmune, Gaithersburg, MD USA; 12 Department of Genetic and Molecular Biology, Hôpital Cochin, AP-HP, 27 rue du Faubourg St Jacques, 75014 Paris, France

**Keywords:** Immunotherapy, PD-1, Interferon, Tumor, Inflammation, Signature

## Abstract

**Background:**

The 18-gene tumor inflammation signature (TIS) is a clinical research assay that enriches for clinical benefit to immune checkpoint blockade. We evaluated its ability to predict clinical benefit of immunotherapy in cancer patients treated with PD-1 checkpoint inhibitors in routine clinical care.

**Methods:**

The CERTIM cohort is a prospective cohort which includes patients receiving immune checkpoint inhibitors in Cochin University hospital. RNA extracted from 58 archival formalin fixed paraffin embedded tumor blocks (including 38 lung cancers, 5 melanomas, 10 renal carcinomas, 4 urothelial carcinomas and 1 colon carcinoma) was hybridized to a beta version of the NanoString^®^ PanCancer IO360™ CodeSet using nCounter^®^ technology. Gene expression signatures were correlated with tumor responses (by RECIST criteria) and overall survival. PD-L1 immunostaining on tumor cells was assessed in 37 non-small cell lung cancer (NSCLC) samples and tumor mutational burden (TMB) measured by whole exome sequencing in 19 of these.

**Results:**

TIS scores were significantly associated with complete or partial response to anti-PD-1 treatment in the whole cohort (odds ratio = 2.64, 95% CI [1.4; 6.0], *p *= 0.008), as well as in the NSCLC population (odds ratio = 3.27, 95% CI [1.2; 11.6], *p *= 0.03). Patients whose tumor had a high TIS score (upper tertile) showed prolonged overall survival compared to patients whose tumor had lower TIS scores, both in the whole cohort (hazard ratio = 0.37, 95% CI [0.18, 0.76], *p *= 0.005) and in the NSCLC population (hazard ratio = 0.36, 95% CI [0.14, 0.90], *p *= 0.02). In the latter, the TIS score was independent from either PD-L1 staining on tumor cells (spearman coefficient 0.2) and TMB (spearman coefficient − 0.2).

**Conclusions:**

These results indicate that validated gene expression assay measuring the level of tumor microenvironment inflammation such as TIS, are accurate and independent predictive biomarkers and can be easily implemented in the clinical practice.

## Background

Immune checkpoint inhibitors (ICI), specifically blockade of the PD-1/PD-L1 pathway via monoclonal antibodies, have entered the oncology therapeutic arsenal and are being evaluated in an increasing number of indications, although the response rates as single agents and in unselected patient populations are usually low. A variety of biomarkers are currently being explored as strategies to enrich for clinical responders, and for some indications, approvals of the anti-PD-1/PD-L1 therapeutic incorporate biomarker testing in the form of PD-L1 measurement by immunohistochemistry (IHC) into the prescribing label. Despite being cleared by the FDA as companion and/or complementary diagnostics for different anti-PD-1/PD-L1 drugs, PD-L1 IHC assays have a number of limitations, including suboptimal positive and negative predictive value and reproducibility. As ICI disrupt pathways that are conserved between tumor cells from multiple different tumor types and the immune environment, biomarkers which are predictive across a variety of indications are of particular interest.

Several biomarkers have been developed which characterize either the potential for tumor cells to prime an adaptive immune response, e.g. tumor mutation burden (TMB) and microsatellite instability (MSI), or the downstream consequences of immune activation, as measured by presence of immune cells e.g. Immunoscore, or gene expression signature related to immune environment [1]. A number of candidate gene expression signatures have been developed, and most focus on biology of activated T cells and IFNγ signaling [2, 3] Interestingly, defects in the IFNγ signaling pathway have been identified as a mechanism of resistance, strongly supporting a crucial role of IFNγ in the anti-cancer immune response [4, 5]. An association between IFNγ expression or IFNγ inducible gene signature and clinical responses to anti-PD-1/PD-L1 mAb was reported in melanoma [6] and bladder carcinomas [7]. Recently an 18 gene “tumor inflammation signature” (TIS) which quantifies an activated but suppressed adaptive immune response in the tumor microenvironment, was demonstrated to retrospectively predict clinical benefit of anti-PD-1 in various cancer types (melanoma, head and neck squamous cell carcinomas, digestive cancers, ovarian and triple negative breast cancers) in clinical trials [2]. The signature has been analytically validated [8, 9] and is currently under investigation in multiple Research Use Only (RUO) and Investigational Use Only (IUO) studies for performance as a predictive biomarker.

In order to assess the feasibility and utility of gene expression profiling in routine clinical practice, we assessed the TIS score from a mixed tumor cohort of patients treated with anti-PD-1 on label at a single center as part of a larger transcriptional profiling study with the PanCancer IO360 gene expression panel.

## Methods

### Patients

Fifty-eight patients from the CERTIM (Immunomodulatory Therapies Multidisciplinary Study group) were included in the study. The CERTIM cohort was initiated in our hospital in July 2015 and prospectively includes all the patients treated with a PD-1 checkpoint inhibitor (either nivolumab or pembrolizumab) administered per label as a single agent, for advanced solid tumor. Treatment was continued until disease progression or intolerable toxicity, physician or patient decision. Tumor imaging by CT scan was performed at baseline, every 8 weeks for patients receiving nivolumab and every 6 weeks for patients receiving pembrolizumab through the first 6 months and every 12 weeks for both thereafter. Response was assessed per Response Evaluation Criteria in Solid Tumors (RECIST) version 1.1. Clinically stable patients considered to be deriving clinical benefit could continue therapy until disease progression was confirmed on imaging done at least 4 weeks after the first assessment of stable disease. The toxicities observed in the patient cohort were monthly reviewed by the dedicated CERTIM multidisciplinary board. Clinical status was assessed monthly during treatment and continued when the treatment was stopped. Database lock-up was 9 February 2018. Median patients follow-up was 19.2 months.

This study was approved by the ethics committee (CPP Ile de France II, no. 2008-133, 2012 06-12, 2018 MS1) in agreement with article L.1121-1 of French law.

### PD-L1 immunostaining

For each tumor, we performed PD-L1 immunostaining on fresh-cut slides from representative FFPE blocks using an anti-PD-L1 antibody (E1L3N, Cell signaling) on Bond automat (Leica) as previously described and validated by the PATTERN French thoracic pathologists group [10]. Staining was blinded analyzed (DD, ALM, BB) on tumoral cells.

### Genomic DNA extraction and illumina-based whole-exome sequencing

Genomic DNA from 22 tumors was isolated from formalin-fixed paraffin-embedded (FFPE) blocks using Maxwell 16 FFPE Tissue LEV DNA Purification Kit (Promega), according to the manufacturer’s instructions. Whole-exome was sequenced as previously reported [11]. DNA were sequenced on the Novaseq 6000 platform, using at least 100 ng of double stranded tumoral DNA (Qubit dsDNA HS kit). After shearing, 11 randomly selected samples were run on the Agilent Bioanalyzer to confirm successful shearing prior to library construction The SureSelect Human All Exon V6 capture kit was used to capture coding regions of genes included in the major genomic databases. Paired end FASTQ files of 101mer sequence reads were generated. All sequence data was quality controlled for read counts, quality values, kmer usage, GC-content, and all other relevant parameters with FastQC (v0.10.1). The DNA read sequences were aligned to the genome (UCSC hg19; Feb 2009 release) using BWA (v0.7.15) and reads sorting and PCR duplicate removal were conducted using Picard (v2.8.3). VarScan2 (v2.4.2) with Samtools mpileup (v1.3) was used to call SNVs/Indels against human reference genome. Germline polymorphisms were removed by retaining only variants with MAF in all races of < 1% or unknown MAF within the 1000 genomes and NHLBI-ESP project with 6500 exome database. The retained SNVs/indels were further filtered by removing SNPs in dbSNP129. Depth of sequencing coverage was 60× on average across all samples in the cohort.

### RNA extraction and hybridization to nCounter codeset

Archival biopsies of patients included in the CERTIM cohort, sampled before anti-PD-1 treatment and with adequate tumor tissue left in FFPE blocks were selected for RNA extraction. RNA was extracted with High Pure FFPE RNA Isolation Kit (Roche) from FFPE tumor samples according to the recommendations of the manufacturer and quantified using fluorimetry with Qubit™ RNA XR Assay Kit (Invitrogen). 100 ng RNA (46 samples) or 30 to 85 ng RNA (12 samples) were successfully hybridized to a beta version of the NanoString^®^ PanCancer IO 360 Panel code set, according to the recommendations of the manufacturer.

### Statistical analysis of transcriptional data

Raw data for each sample and gene were normalized to internal ERCC controls to eliminate technical variability of the assay, and then counts were normalized to the geometric mean of endogenous housekeeping genes followed by log2 transformation. Gene expression signatures, including the TIS, were calculated as a weighted linear average of the constituent genes [2, 12]. The weighted scores used for calculation of the TIS and other signatures are NanoString intellectual property. For the correlation analysis with clinical response to treatment, clinical benefit was defined as complete or partial RECIST response while stable and progressive disease were defined as lack of clinical benefit. Normalized gene counts and signature scores were compared to the response category using a linear model. The log2 fold change, Wald-type confidence interval and *p*-value were calculated for each gene and signature (Additional file 1: Table S1 and Additional file 2: Table S2). To assess predictive performance of the additional signatures above and beyond the predictive performance of TIS, a logistic regression model was used to assess performance conditional upon TIS score.

For the analysis on the survival time, the genes and scores were dichotomized into high and low groups based on median, with the exception of TIS which was divided into tertiles. The survival time was fit to the binary group with Cox proportional hazard model. The hazard ratio between the high and low group, the Wald-type confidence interval and the log-rank test *p*-value are reported for each gene and signature (Additional file 1: Table S1 and Additional file 2: Table S2).

## Results

### Tumor inflammation signature enriches for clinical response to anti-PD-1 in the multi-tumor cohort

**Fig. 1:**
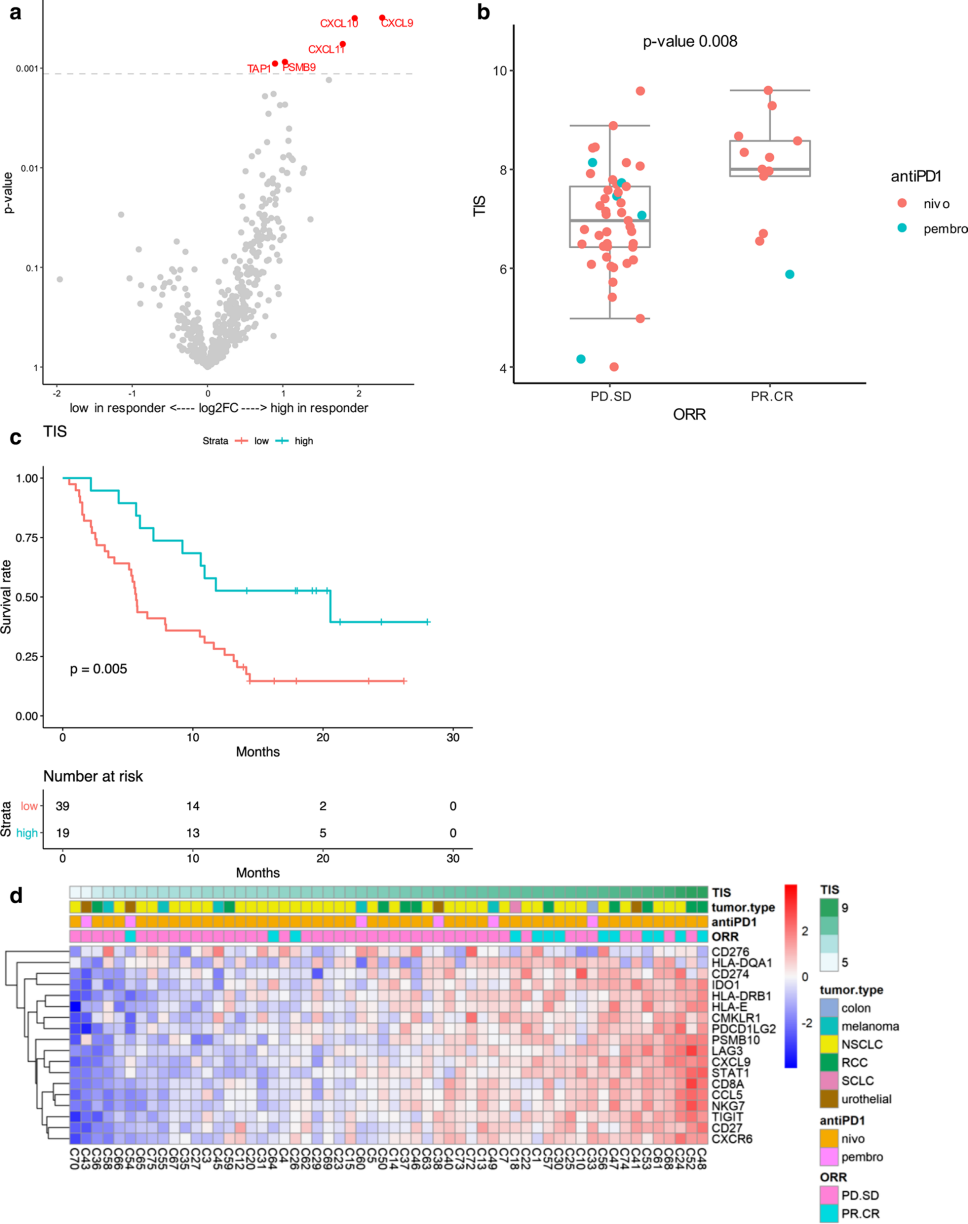
TIS scores are associated with response to anti-PD-1 in the CERTIM multi-tumor cohort. **a** Volcano plot of *p*-value versus log2 fold change of the differential expression between responders (PR/CR) and non-responders (PD/SD) in the whole cohort. The test for differential expression was done by fitting the log2 normalized count to the response with linear model. The *p*-values were adjusted by the Benjamini–Hochberg Procedure. Dots corresponding to genes that are significant at FDR < 0.1 are labelled in red. **b** Boxplot of TIS scores in responders and non-responders (The lower whisker of the responders is not visible as the length is 0). The response was fit to TIS scores with logistic regression and *p*-value = 0.008, indicating that high TIS scores are predictive of response to anti-PD-1 treatment. The odds ratio is 2.64, 95% confidence interval (1.37, 5.95). **c** The Kaplan–Meier curves of TIS score groups for the whole patient cohort. The TIS scores were categorized into three groups by tertiles. The Kaplan–Meier curves show that the high TIS score group have higher survival rate than the other two groups (which are combined on the graph into the “low” group). The survival time was fit to TIS score group (high vs low and intermediate) with Cox proportional hazard model. Hazard ratio is 0.374, 95% confidence interval (0.18, 0.76), *p*-value = 0.005, meaning the high TIS score group has a decrease of the hazard by 63%. **d** Heatmap showing the individual TIS genes normalized expression, as well as TIS global score, histological subtype and overall response to anti-PD1. *NSCLC* non small cell lung carcinoma, *RCC* renal cell carcinoma, *SCLC* small cell lung carcinoma, *nivo* nivolumab, *pembro* pembrolizumab, *ORR* overall response according to RECIST v1.1, *CR* complete response, *PR* partial response, *SD* stable disease, *PD* progressive disease

Consecutive metastatic cancer patients treated with anti-PD-1 monoclonal antibodies in the outpatient monocentric CERTIM cohort with available FFPE tumor specimen were selected for analysis of gene expression and immune signatures using NanoString PanCancer IO 360 Panel (beta version), which contains an RUO version of the TIS. The clinical characteristics of the 58 patients with different cancer types included in this study are described in Table 1. Several genes showed a differential expression in responders to anti-PD-1 [patients with complete response (CR) or partial response (PR) according to RECIST criteria] compared to non-responders (stable or progressive disease) (Additional file 1: Table S1). After correction for multiple testing, 5 genes related to IFNγ signaling and antigen processing remained significantly higher in responders: *CXCL9*, *CXL10*, *CXCL11*, *TAP1* and *PSMB9* (Fig. 1a). Since a number of the genes with greatest association with clinical benefit are contained within or closely related to genes in the TIS, we evaluated the TIS as a predictive biomarker in this cohort. In this study, a high TIS score was significantly associated with response to anti-PD-1 treatment (odds ratio = 2.64, 95% CI [1.4; 6.0], *p *= 0.008, Logistic regression) (Fig. 1b). Furthermore, patients with a high TIS score (upper tertile) had a prolonged overall survival compared to patients with lower scores (hazard ratio = 0.37, 95% CI [0.18, 0.76], *p*-value = 0.005, Cox regression) (Fig. 1c). The expression of 16 genes included in the TIS score were closely correlated, whereas *CD276* and *HLA*-*DQA1* expression appeared more variable across TIS scores (Fig. 1d). The normalized gene expression data, TIS score, as well as response to ICI and survival for each of the samples included in this study are provided in Additional file 3: Table S3. Altogether, these data indicate that the TIS is significantly associated with clinical benefit of anti-PD-1 (pembrolizumab or nivolumab) in a « real life » cohort of patients.

**Table 1:** Clinical characteristics of the patients in the CERTIM multi-cancer cohort

	*N* (%)
Sex	
M	38 (66%)
F	20 (44%)
Age (year)	
Median (range)	66 (41–83)
Tumor type	
Non-small cell lung	37 (64%)
Small cell lung	1 (2%)
Melanoma	5 (9%)
Colon	1 (2%)
Renal	10 (17%)
Urothelial	4 (7%)
ECOG performance status^a^	
0	2 (3%)
1	31 (54%)
≥ 2	24 (42%)
Previous lines of therapy	
0	2 (3%)
1	31 (53%)
2	11 (19%)
≥ 3	14 (24%)
Anti-PD-1	
Nivolumab	52 (90%)
Pembrolizumab	6 (10%)
ORR	
CR	6 (10%)
PR	7 (12%)
SD	7 (10%)
PD	38 (65%)

^a^ECOG status was not available for 1 patient

### TIS predictive of anti-PD-1 benefit in non small cell lung cancer (NSCLC) cohort

**Fig. 2:**
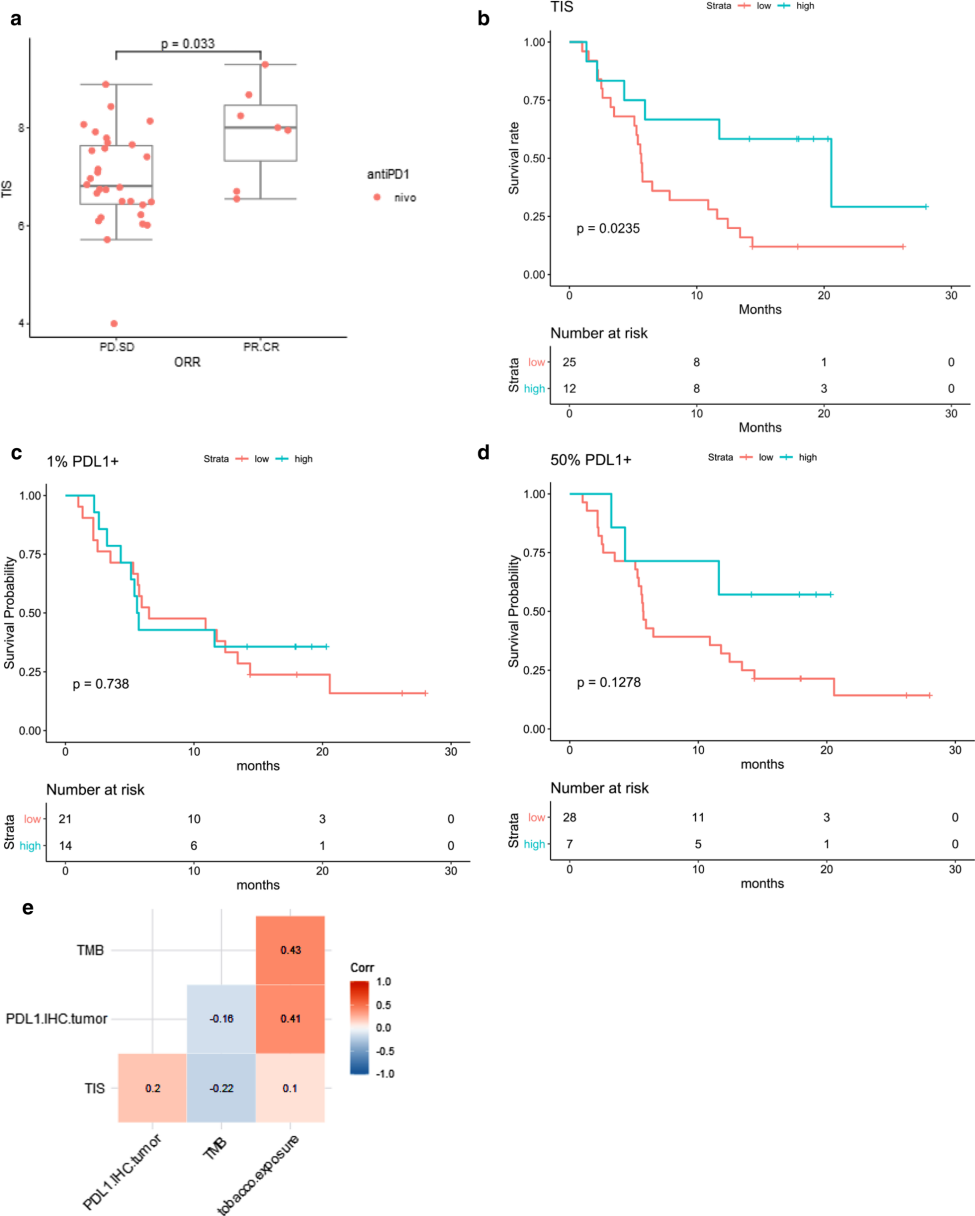
Performance of TIS assay vs other biomarkers in NSCLC cohort. **a** Boxplot of TIS scores in responders and non-responders in the NSCLC cohort. All patients were treated with nivolumab. The response was fit to TIS scores with logistic regression and *p*-value = 0.033, indicating that high TIS scores are predictive of tumor response to anti PD-1 treatment. The odds ratio is 3.27, 95% confidence interval (1.23, 11.63). **b** The Kaplan–Meier curves of TIS score groups for the NSCLC cohort. Patients are stratified by TIS score tertiles, and the highest TIS score group was observed to have longer survival than the other two groups (which are combined into the “low” group on the graph). The survival time is fit to TIS score group (high vs low and intermediate) with Cox proportional hazard model. Hazard ratio is 0.36, 95% confidence interval (0.14, 0.90), *p*-value = 0.0235, meaning the high TIS score group has a decrease of the hazard by 64%. **c** The Kaplan–Meier curves of NSCLC patients with 1% PD-L1 positivity on tumor cells used as a cutoff. The survival time in the NSCLC cohort is fit to PD-L1+ group (high vs low) with Cox proportional hazard model. **d** The Kaplan–Meier curves of NSCLC patients with 50% PD-L1 positivity on tumor cells used as a cutoff. The survival time in the NSCLC cohort is fit to PD-L1+ group (high vs low) with Cox proportional hazard model. Analysis suggests that patients with 50% PD-L1+ tumor cells may have longer survival, but the sample size is too limited to reach statistical significance. **e** The Spearman correlation matrix between TIS scores, percentage of PD-L1+ tumor cells, tumor mutation burden and tobacco exposure for NSCLC cohort. PD-L1+ tumor cells and TMB are positively correlated with tobacco exposure

We then focused our analysis on NSCLC which represented the majority of the cases that were studied in this cohort. All 37 patients had received nivolumab, and the clinical characteristics of the patients, including the tumor subtype and smoking status, are indicated in Table 2. Overall, 7/37 (19%) patients responded to treatment. As in the whole cohort, we observed that TIS enriched for tumor response in NSCLC (odds ratio = 3.27, 95% CI [1.2; 11.6], *p *= 0.03, Logistic regression) (Fig. 2a). Furthermore, patients with a high TIS score (upper tertile) had a prolonged survival compared to those with lower TIS scores (hazard ratio = 0.36, 95% CI [0.14, 0.90], *p*-value = 0.02, Cox regression) (Fig. 2b). In order to assess the predictive value of TIS score within the context of one of other biomarkers of clinical interest, we also scored the NSCLC samples for PD-L1 expression by IHC at the 1% and 50% cutoff for tumor cell positivity, the two cut offs reported in the label for second and first line NSCLC patient selection for treatment with pembrolizumab. PD-L1 staining ≥ 1% tumor cells was not significantly associated with survival (hazard ratio = 0.87, CI [0.4, 2], *p *= 0.74), and PD-L1 staining on 50% tumor cells had a trend with overall survival but did not reach statistical significance (hazard ratio = 0.40, CI [0.1, 1.3], *p *= 0.13) (Fig. 2c, d). Additionally, tumor mutational burden by whole exome sequencing was available for 19 of the patients (see Additional file 4: Table S4 for baseline characteristics). The tumor mutational burden ranged from 13.7 to 26.8 mutations/MB and was positively correlated with the number of smoking pack years (spearman coefficient 0.43). In this limited patient cohort, TMB was lower in adenocarcinoma (n = 11, median = 20.3 mutation/Mb) than in squamous cell carcinoma (n = 6, median = 23.3 mutation/Mb) (*p* value = 0.01, Fisher test), and was not significantly associated with survival (hazard ratio = 1.91, CI [0.6, 6.2], *p *= 0.25). In this small cohort, TIS was still significantly associated with overall survival (p = 0.02, data not shown). Finally, we assessed whether any of the biomarkers were associated with one another, and observed that PD-L1 staining on tumor cells and TMB were positively correlated with tobacco exposure, but the other biomarkers were not strongly associated with each other (Fig. 2e). Specifically, PD-L1 IHC staining was not significantly with TMB (spearman coefficient − 0.16, p value 0.53), and the TIS was not significantly correlated with either PD-L1 immunohistochemical staining (spearman coefficient 0.20, p value 0.25), or TMB (spearman coefficient − 0.22, p value 0.38).

**Table 2:** Clinical characteristics of the patients in the CERTIM NSCLC cohort

Characteristic	Category	N (%)
Sex	M	23 (62%)
	F	14 (38%)
Age (year)	Median (range)	68 (41–78)
Tumor type	Adenocarcinoma	25 (68%)
	Squamous cell carcinoma	10 (27%)
	NOS	2 (5%)
Smoking status	Non smoker	4 (11%)
	Smokers	33 (88%)
	< 10 pack/year (≤ 10 packs years)	–
	[10–30] pack/year	19 (57%)
	> 30 pack/year	14 (42%)
	Quit > 1 year	19 (57%)
	Active or quit ≤ 1 year	14 (42%)
ECOG performance status	0	1 (3%)
	1	18 (49%)
	≥ 2	18 (49%)
Previous lines of therapy	0	–
	1	22 (59%)
	2	6 (16%)
	≥ 3	9 (24%)
ORR	CR	3 (8%)
	PR	4 (11%)
	SD	6 (16%)
	PD	24 (65%)

### Additional signatures beyond TIS predictive of anti-PD-1 benefit

**Fig. 3:**
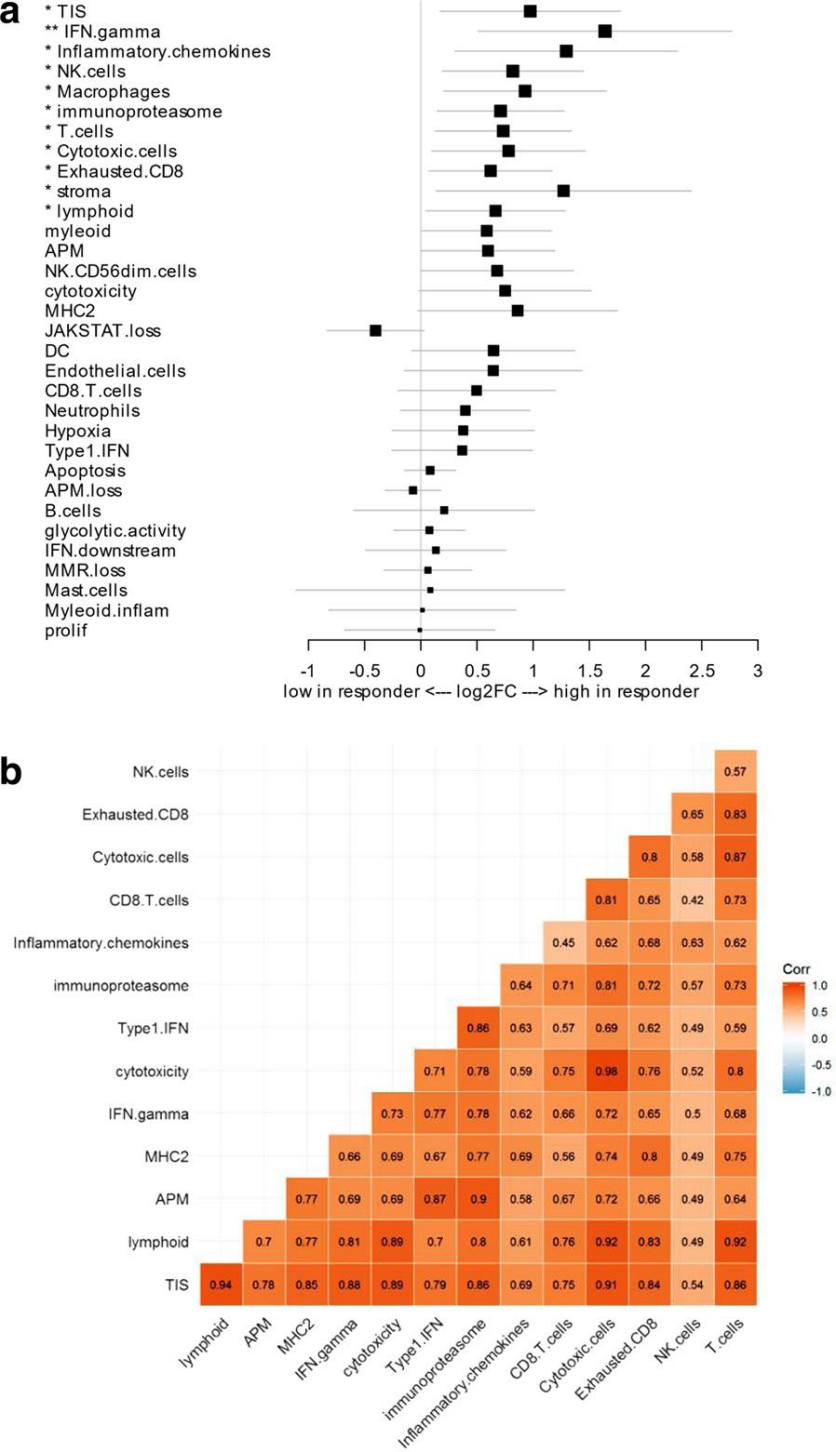
Additional gene expression signatures associated with clinical benefit of anti-PD-1 in NSCLC. **a** Forest plot of difference of multi-gene signature scores between responders and non-responders in the NSCLC cohort. The position of the squared dots denotes the difference of score, and the size denotes the statistical significance. The horizontal lines are the Wald-type confidence intervals. The * sign denotes the significance of *p*-value (< 0.001***, < 0.01**, < 0.05*). **b** The Spearman correlation matrix between the signature scores was calculated for the signatures that showed significant difference in the differential expression analysis between responder and non-responders

We next evaluated a number of other predefined immune gene signatures contained within the IO360 panel for their association with response in the NSCLC cohort and identified several signatures that were statistically significant, including IFNγ signaling, lymphoid cells, T cell, NK cells, cytotoxic cells, exhausted CD8 T cells, macrophages, stroma, inflammatory chemokines, and immunoproteasome (Fig. 3a). The majority had strong positive correlation with TIS and with each other (Fig. 3b). Multivariate analysis showed that none of these gene signatures significantly predicted for response after correcting for TIS (data not shown). The signatures were then evaluated to see which were also predictive of overall survival in the NSCLC cohort, and only TIS, stroma, and lymphoid signatures were significantly associated (Additional file 5: Figure S1). In the multi-tumor cohort, several signatures were associated with response, but only IFNγ signature remained significant after adjusting for TIS (*p*-value = 0.03) (Additional file 6: Figure S2A); whereas TIS, immunoproteasome, lymphoid and hypoxia signatures were predictive of survival (Additional file 6: Figure S2B). These findings indicate that TIS is a robust biomarker of both response and survival following PD-1 checkpoint blockade in both NSCLC and multi-tumor cohorts from real world patients.

## Discussion

The limited efficacy, potential toxicity and the cost of anti-PD-1/PD-L1 molecules in cancers patients calls for the use of predictive biomarkers. Our study focused on gene expression based biomarkers because they are compatible with current clinical practice, i.e. they can be performed with small tissue samples and using a local laboratory and have the potential for clinical utility across disease indications and therapeutic agents. The TIS was originally described as a biomarker predictive of response in patients with different cancer types, treated with pembrolizumab in the context of clinical trials [2]. Here, we extend the characterization of TIS to include novel indications (NSCLC) and novel agents (nivolumab). One strength of this study is that it was performed on patients treated in the community setting; thus in a less selected patient population than in clinical trials and likely more reflective of larger real world performance. This study demonstrates an association of high TIS with ICI benefit, for both clinical response and overall survival, independently of carcinoma subtype and anti-PD-1 therapeutic molecule administered.

The study has a number of limitations which must be considered in the interpretation of results. First, the study is composed of a small cohort of patients, especially the lung cancer cohort. Despite the small cohort size, the TIS was predictive of both response and survival, and thus may represent a realistic option for the identification of patients who may benefit from ICI. We await the availability of larger cohorts of patients treated with ICI in the real life management to confirm these results. Ultimately, prospective clinical trials using TIS as a stratifying biomarker are needed to definitely confirm its predictive value in different cancer types.

It is worth considering the performance of the non-gene signature biomarkers in this study. Although it is the approved diagnostic, PD-L1 IHC was not observed to be predictive, which may be due to tissue heterogeneity and the cohort size. Small biopsies sometimes comprise small numbers of tumor cells and the positivity of these cells may not reflect the whole tumor status [13]. Using multigene signatures such as the TIS may be a more robust way of measuring the presence of a cytotoxic anti-tumor immune response than measuring PD-L1 protein alone. Similarly, high TMB has been shown to be associated with ICI benefit, particularly in lung cancer, in numerous studies [14–17], but it was not predictive in this cohort of NSCLC, nor was it correlated with TIS. This may be due to the limited number of cases studied, mixed histology (adenocarcinoma and SCC) and/or the fact that TMB was high in all samples (13.7 to 26.8 mutations/MB). Interestingly, a recent study established that TMB and TIS were independently predictive of clinical response to pembrolizumab in KEYNOTE clinical trials in a variety of tumors but without examining NSCLC [18]. Further limiting its utility, at the current time TMB measurement is a costly technique, and is awaiting for international standardization and recommendations [19].

Finally, gene signatures beyond TIS may be required in the future to further dissect mechanisms of immune resistance in patients whose tumor does not respond to single agent PD-1/PD-L1 blockade to inform biology-based combinations. Specifically, while tumor inflammation gene signatures, including TIS, are measuring IFN biology and/or presence of specific T cell populations [2, 6, 20–22], additional signatures (which could be combined with TIS) are needed to gain further insight into additional processes associated with immune escape that could be targeted therapeutically [23].

## Conclusions

In this study, gene expression signatures were analyzed in a cohort of FFPE tumor samples from cancer patients treated with anti-PD-1 in routine clinical care. The tumor inflammation signature was significantly associated with clinical response and overall survival, supporting its evaluation in parallel with other biomarkers in routine practice and future clinical trials.

## Supplementary information

**Additional file 1: Table S1.** Statistical correlation of individual gene expression with response and survival in the whole cohort and in the NSCLC cohort.

**Additional file 2: Table S2.** Statistical correlation of gene signatures with response and survival in the whole cohort and in the NSCLC cohort.

**Additional file 3: Table S3.** Histological subtype, clinical outcome (response to anti-PD1 and survival), TIS score and normalized gene expression data, for each of the 58 samples included in the study.

**Additional file 4: Table S4.** Clinical characteristics of the NSCLC patients with TMB data.

**Additional file 5: Figure S1.** Immune signatures associated with overall survival in the NSCLC cohort. Forest plot of hazard ratio in the survival analysis between high and low signature score in the NSCLC cohort. The signature scores are dichotomized into high and low groups by their median (except TIS scores uses the upper tertile as in Fig. 2b). The survival time is fit to score group (high vs low) with Cox proportional hazard model. The hazard ratio and Wald-type confidence interval are estimated. The *p*-value are determined by the log-rank test. The * sign denotes the significance of *p*-value (< 0.001***, < 0.01**, < 0.05*).

**Additional file 6: Figure S2.** TIS scores and other gene signatures associated with response and overall survival in the CERTIM multi-tumor cohort. **a** Forest plot of difference of multi-gene signature scores between responders and non-responders in the CERTIM multi-tumor cohort. The position of the squared dots denotes the difference of score, and the size denotes the statistical significance. The horizontal lines are the Wald-type confidence intervals. The * sign denotes the significance of *p*-value (< 0.001***, < 0.01**, < 0.05*). **b** Forest plot of hazard ratio in the survival analysis between high and low signature score in the CERTIM cohort. The signature scores are dichotomized into high and low groups by their median (except TIS scores uses the upper tertile as in Fig. 1c). The survival time is fit to score group (high vs low) with Cox proportional hazard model. The hazard ratio and Wald-type confidence interval are estimated. The *p*-value are determined by the log-rank test. The * sign denotes the significance of *p*-value (< 0.001***, < 0.01**, < 0.05*).

## Data Availability

Not applicable.

## Abbreviations

CERTIM: Immunomodulatory Therapies Multidisciplinary Study group
CR: complete response
FFPE: formalin-fixed paraffin-embedded
ICI: immune checkpoint inhibitors
IHC: immunohistochemistry
IUO: Investigational Use Only
MSI: microsatellite instability
NSCLC: non-small cell lung cancer
PR: partial response
RECIST: Response Evaluation Criteria in Solid Tumors
RUO: Research Use Only
TIS: tumor inflammation signature
TMB: tumor mutational burden
