# Supplementary material for: The tumor inflammation signature (TIS) is associated with anti-PD-1 treatment benefit in the CERTIM pan-cancer cohort
Source: J Transl Med. 2019 Nov 4;17:357. doi: 10.1186/s12967-019-2100-3 (PMC6829827; doi:10.1186/s12967-019-2100-3)
Supplement: Supplementary file 4 — Additional file 4: Table S4. Clinical characteristics of the NSCLC patients with TMB data. [file 12967_2019_2100_MOESM4_ESM.docx]

**Supplemental Table 4. Clinical characteristics of the patients with NSCLC and WES data**

| **Characteristic** | **Category** | **N (%)** |
| --- | --- | --- |
| **Sex** |  |  |
|  | M | 13 (68%) |
|  | F | 6 (32%) |
| **Age – year** |  |  |
|  | Median (range) | 68 (41-78) |
| **Tumor Type** |  |  |
|  | Adenocarcinoma | 11 (58%) |
|  | Squamous cell carcinoma | 6 (32%) |
|  | NOS | 2 (11%) |
| **Smoking Status** |  |  |
|  | Non smoker | 2 (11%) |
|  | Smokers | 17 (89%) |
|  | *< 10 Pack/Year*  *(≤10 packs years)* | - |
|  | *[10-30] Pack/Year* | 7 (41%) |
|  | *> 30 Pack/Year* | 10 (59%) |
|  | *Quit >1 year* | 11 (65%) |
|  | *Active or quit ≤ 1 year* | 6 (35%) |
| **ECOG Performance Status** |  |  |
|  | 0 | 1 (5%) |
|  | 1 | 9 (47%) |
|  | ≥ 2 | 9 (47%) |
| **Previous Lines of Therapy** |  |  |
|  | 0 | - |
|  | 1 | 12 (63%) |
|  | 2 | 4 (21%) |
|  | ≥3 | 3 (16%) |
| **ORR** |  |  |
|  | CR | 1 (5%) |
|  | PR | 2 (11%) |
|  | SD | 5 (26%) |
|  | PD | 11 (58%) |
