# Supplementary figures and images for: The tumor inflammation signature (TIS) is associated with anti-PD-1 treatment benefit in the CERTIM pan-cancer cohort
Source: J Transl Med. 2019 Nov 4;17:357. doi: 10.1186/s12967-019-2100-3 (PMC6829827; doi:10.1186/s12967-019-2100-3)

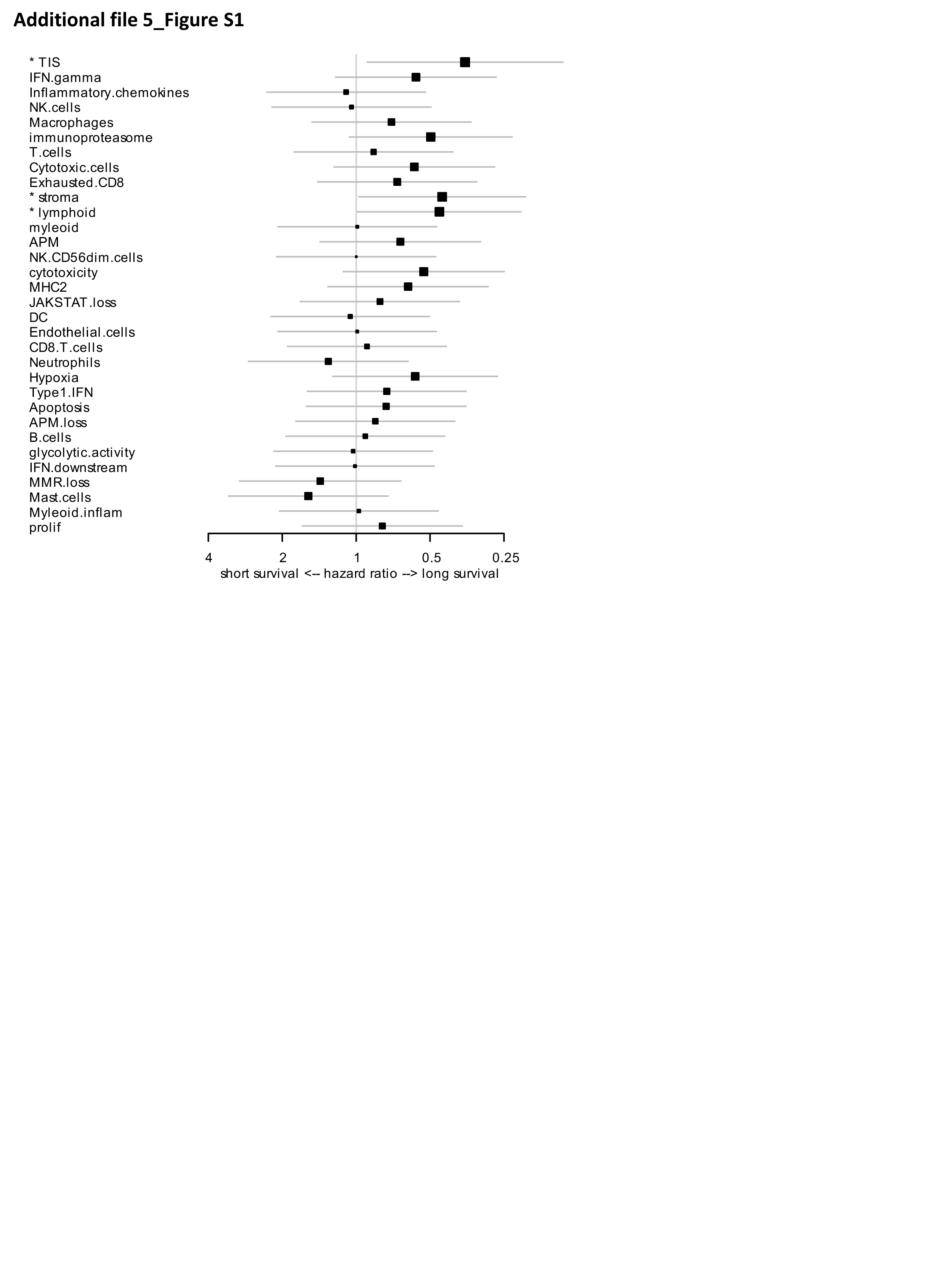

Supplement: Supplementary file 5 — Additional file 5: Figure S1. Immune signatures associated with overall survival in the NSCLC cohort. Forest plot of hazard ratio in the survival analysis between high and low signature score in the NSCLC cohort. The signature scores are dichotomized into high and low groups by their median (except TIS scores uses the upper tertile as in Fig. 2b). The survival time is fit to score group (high vs low) with Cox proportional hazard model. The hazard ratio and Wald-type confidence interval are estimated. The p-value are determined by the log-rank test. The * sign denotes the significance of p-value (< 0.001***, < 0.01**, < 0.05*). [file 12967_2019_2100_MOESM5_ESM.tif]

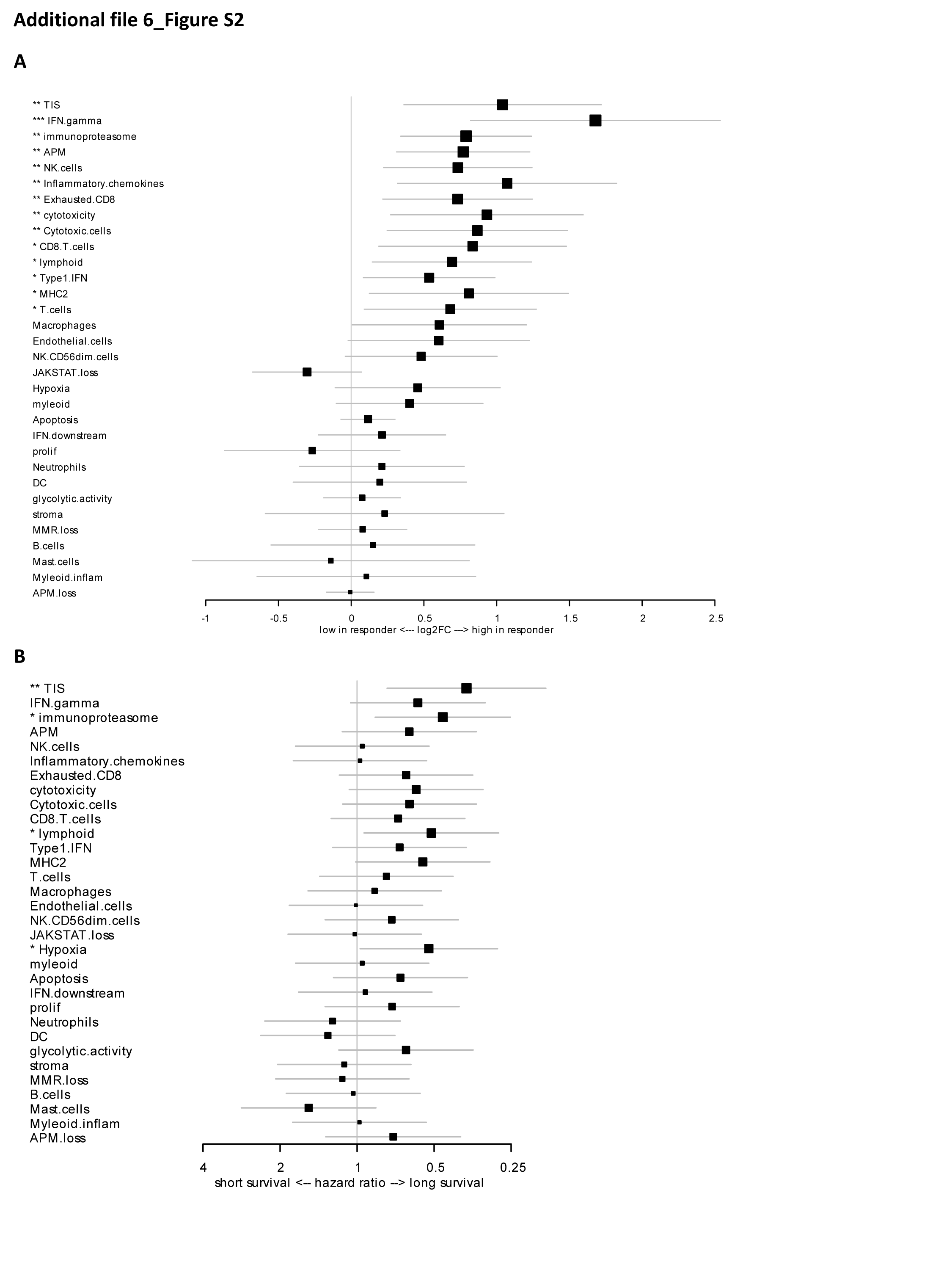

Supplement: Supplementary file 6 — Additional file 6: Figure S2. TIS scores and other gene signatures associated with response and overall survival in the CERTIM multi-tumor cohort. a Forest plot of difference of multi-gene signature scores between responders and non-responders in the CERTIM multi-tumor cohort. The position of the squared dots denotes the difference of score, and the size denotes the statistical significance. The horizontal lines are the Wald-type confidence intervals. The * sign denotes the significance of p-value (< 0.001***, < 0.01**, < 0.05*). b Forest plot of hazard ratio in the survival analysis between high and low signature score in the CERTIM cohort. The signature scores are dichotomized into high and low groups by their median (except TIS scores uses the upper tertile as in Fig. 1c). The survival time is fit to score group (high vs low) with Cox proportional hazard model. The hazard ratio and Wald-type confidence interval are estimated. The p-value are determined by the log-rank test. The * sign denotes the significance of p-value (< 0.001***, < 0.01**, < 0.05*). [file 12967_2019_2100_MOESM6_ESM.tif]
